# Co-evolution of *AR* gene copy number and structural complexity in endocrine therapy resistant prostate cancer

**DOI:** 10.1093/narcan/zcad045

**Published:** 2023-08-24

**Authors:** Andrej Zivanovic, Jeffrey T Miller, Sarah A Munro, Todd P Knutson, Yingming Li, Courtney N Passow, Pijus Simonaitis, Molly Lynch, LeAnn Oseth, Shuang G Zhao, Felix Y Feng, Pernilla Wikström, Eva Corey, Colm Morrissey, Christine Henzler, Benjamin J Raphael, Scott M Dehm

**Affiliations:** Masonic Cancer Center, University of Minnesota, Minneapolis, MN, USA; Minnesota Supercomputing Institute, University of Minnesota, Minneapolis, MN, USA; Minnesota Supercomputing Institute, University of Minnesota, Minneapolis, MN, USA; Minnesota Supercomputing Institute, University of Minnesota, Minneapolis, MN, USA; Masonic Cancer Center, University of Minnesota, Minneapolis, MN, USA; University of Minnesota Genomics Center, University of Minnesota, Minneapolis, MN, USA; Department of Computer Science, Princeton University, Princeton, NJ, USA; Masonic Cancer Center, University of Minnesota, Minneapolis, MN, USA; Masonic Cancer Center, University of Minnesota, Minneapolis, MN, USA; Department of Human Oncology, University of Wisconsin-Madison, Madison, WI, USA; Carbone Cancer Center, University of Wisconsin-Madison, Madison, WI, USA; William S. Middleton Memorial Veterans Hospital, Madison, Madison, WI, USA; Departments of Radiation Oncology, Urology, and Medicine, University of California San Francisco, San Francisco, CA, USA; Helen Diller Family Comprehensive Cancer Center, University of California at San Francisco, San Francisco, CA, USA; Department of Medical Biosciences, Pathology, Umeå University, Umeå, Sweden; Department of Urology, University of Washington, Seattle, WA, USA; Department of Urology, University of Washington, Seattle, WA, USA; Minnesota Supercomputing Institute, University of Minnesota, Minneapolis, MN, USA; Department of Computer Science, Princeton University, Princeton, NJ, USA; Masonic Cancer Center, University of Minnesota, Minneapolis, MN, USA; Department of Laboratory Medicine and Pathology, University of Minnesota, Minneapolis, MN, USA; Department of Urology, University of Minnesota, Minneapolis, MN, USA

## Abstract

Androgen receptor (AR) inhibition is standard of care for advanced prostate cancer (PC). However, efficacy is limited by progression to castration-resistant PC (CRPC), usually due to AR re-activation via mechanisms that include *AR* amplification and structural rearrangement. These two classes of *AR* alterations often co-occur in CRPC tumors, but it is unclear whether this reflects intercellular or intracellular heterogeneity of *AR*. Resolving this is important for developing new therapies and predictive biomarkers. Here, we analyzed 41 CRPC tumors and 6 patient-derived xenografts (PDXs) using linked-read DNA-sequencing, and identified 7 tumors that developed complex, multiply-rearranged *AR* gene structures in conjunction with very high *AR* copy number. Analysis of PDX models by optical genome mapping and fluorescence *in situ* hybridization showed that *AR* residing on extrachromosomal DNA (ecDNA) was an underlying mechanism, and was associated with elevated levels and diversity of AR expression. This study identifies co-evolution of *AR* gene copy number and structural complexity via ecDNA as a mechanism associated with endocrine therapy resistance.

## Graphical Abstract

**Graphical Abstract:**
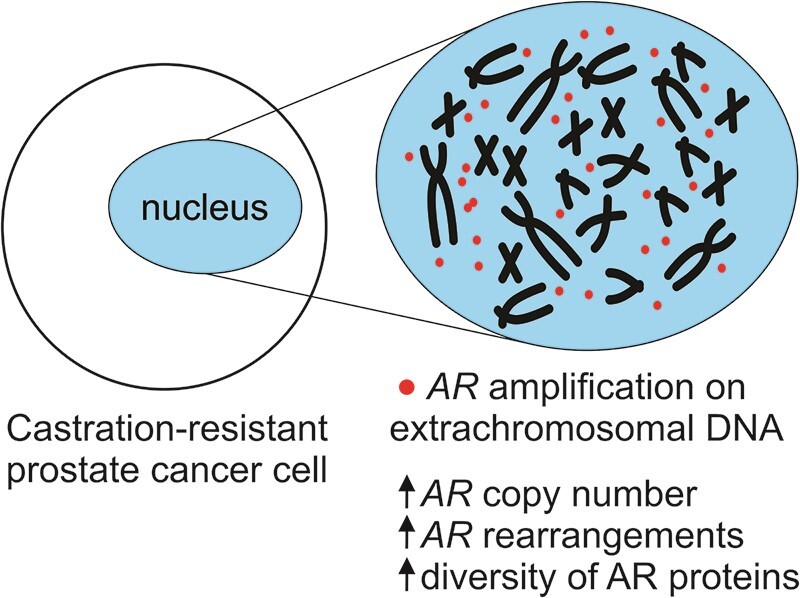

## INTRODUCTION

The androgen receptor (AR) is a nuclear receptor transcription factor activated by androgenic ligands such as testosterone. In the prostate, active AR regulates the expression of genes vital for epithelial cell homeostasis. This AR dependence remains a hallmark of prostate cancer (PC) cells. Therefore, systemic therapies for advanced PC include endocrine therapies that prevent testicular androgen production, the CYP17A1 inhibitor abiraterone that prevents systemic androgen synthesis, and high-affinity competitive AR antagonists such as enzalutamide, apalutamide, and darolutamide (1–5). Despite this repertoire of AR inhibitors, development of a resistance phenotype termed castration resistant PC (CRPC) is frequent. CRPC is a lethal disease stage because combining or switching between AR inhibitors provides little benefit, and few alternative therapies exist (3). However, the observation that CRPC progression is usually marked by rising prostate specific antigen, encoded by the AR-activated *KLK3* gene, indicates that AR transcriptional activity persists in most CRPC tumors and remains an attractive therapeutic target (6).

Analysis of CRPC metastases using short-read DNA-sequencing (DNA-seq) has identified *AR* gene alterations in 75–90% of patients, consisting of amplification, single nucleotide variants (mutations) and structural rearrangements (7–9). Conversely, in therapy-naïve (castration sensitive) PCs, *AR* gene alterations are rare. *AR* amplification and mutation can facilitate AR activation by castrate levels of androgens and convert AR antagonists to agonists (6). However, the role of *AR* gene rearrangements is incompletely understood, which can be attributed to their heterogeneity in CRPC tissues and circulating tumor DNA (ctDNA) (9–14). *AR* gene rearrangements are DNA structural variants (duplications, deletions, inversions, and translocations) with breakpoints that occur within the *AR* gene body, resulting in altered *AR* gene architecture. In 5–10% of CRPC cases, *AR* gene rearrangements exist as the only *AR* alteration detectable, and in this context they promote synthesis of tumor-unique AR variants (AR-V) that lack the AR ligand binding domain and possess constitutive activity that supports growth of CRPC cells (9,13,15). Conversely, 20–25% of CRPC cases harbor *AR* gene rearrangements concurrent with *AR* amplification, often with multiple *AR* gene rearrangement events in a tumor. Due to the limitations of short-read DNA-sequencing (DNA-seq), it remains unclear whether this reflects intercellular or intracellular heterogeneity of *AR* gene rearrangements. Resolving this question is important for developing new therapeutic regimens and biomarkers to ensure elimination of all cells in a CRPC tumor.

In this study, we addressed these considerations by performing targeted linked-read DNA-seq, which is a technique that allows *in silico* reconstruction of original high molecular DNA molecules from which DNA-seq reads were derived (16). We analyzed a cohort of 6 PDXs and 23 CRPC metastases, and also analyzed whole-genome linked-read DNA-seq data from a cohort of 18 CRPC metastases (17). We found that 15% of these tumors displayed very high *AR* copy number as well as complex, multiply-rearranged *AR* gene structures. Complex *AR* gene rearrangements displayed co-evolution with *AR* copy number due to *AR* being captured on double-minute or extrachromosomal DNA (ecDNA), which are large circular DNA molecules in cancer cells known to harbor amplified oncogenes (18). Overall, this study demonstrates that in a subset of CRPC patients, tumor cells can accumulate very high numbers of structurally diverse *AR* gene copies under pressure of AR-targeted therapy, and implicates ecDNA as an underlying mechanism.

## MATERIALS AND METHODS

### Cell lines

The 22Rv1 cell line (CRL-2505) was obtained from American Type Culture Collection (ATCC) (Manassas, VA, USA) in 2013 and cultured in RPMI 1640 medium (Invitrogen) supplemented with 10% fetal bovine serum (FBS) and 1× (100 μg/ml) penicillin/streptomycin. The CWR-R1 late subline was generated from the CWR-R1 cell line as described (19,20), and cultured in RPMI 1640 medium with 10% charcoal stripped (CS) FBS and 1× penicillin/streptomycin. The LNCaP95 cell line was provided by Jun Luo (John Hopkins University) in 2012, and cultured in phenol red-free RPMI 1640 medium (Invitrogen) supplemented with 10% FBS and 1× penicillin/streptomycin. The VCaP cell line (CRL-2876) was obtained from ATCC in 2011, and cultured in DMEM medium (Invitrogen) + 10% FBS and 1× penicillin/streptomycin.

### Patient-derived xenografts

Patient-derived xenograft (PDX) models LuCaP 86.2, LuCaP 35, LuCaP 35CR, LuCaP 77, LuCaP 77CR, LuCaP 105 and LuCaP 105CR have been described (21). LuCaP 86.2, LuCaP 35, LuCaP 77 and LuCaP 105 tumors were propagated in intact CB17 SCID mice (Charles River). LuCaP 35CR, LuCaP 77CR, and LuCaP 105CR tumors were propagated in CB17 SCID castrated mice. All mouse protocols were approved by the University of Minnesota and University of Washington Institutional Care and Use Committees (IACUC).

### *Ex vivo* culture of PDX-derived cells

PDXs were resected from mice, cut into pieces with dimensions of 2–4 mm, and dissociated enzymatically using a human tumor dissociation kit (Miltenyi Biotec) and a tissue dissociator (Miltenyi Biotec) per manufacturer's specifications. Red blood cells were lysed with ammonium chloride potassium (ACK) buffer (Lonza). The cells were passed through 100 and 40 μm strainers (Falcon) sequentially to filter coarse particulates. Cultures were established by plating cells in prostate-specific organoid medium without dihydrotestosterone in 6-well tissue culture dishes (Falcon) without matrigel for five days.

### Clinical specimens

Metastatic CRPC samples labeled 00 through 14 were obtained from patients who died of CRPC from 2000 through 2014 and signed written informed consent for a rapid autopsy under the aegis of the Prostate Cancer Donor Program at the University of Washington (IRB protocol no. 2341) (22–24). Metastatic CRPC samples starting with ‘T’ were obtained during palliative surgery for bone metastases at the University Hospital of Umea, Sweden (25). Patients gave their informed consent and the study was approved by the local ethic review board of Umeå University (Dnr 03–158, Dnr 2013-57-31M with amendment Dnr 2016–313-32M).

### AR-targeted linked-read DNA-sequencing (DNA-seq)

Genomic DNA (gDNA) was isolated from cell lines and tissues using a Nucleospin Triprep kit (Macherey-Nagal). DNA was submitted to the University of Minnesota Genomics Center for linked-read DNA-seq library preparation and barcoding using the Chromium platform (10× Genomics). In brief, gDNA was diluted to 0.8–1.2 ng/μl, and 10 μl of diluted gDNA was added to a Sample Master Mix using the Chromium Genome Reagent Kits with beads (10× Genomics). The Sample Master Mix was loaded onto a genome chip, along with genome gel beads and partitioning oil and loaded on a Chromium controller. Once complete, the gel-bead in emulsions (GEMs) were transferred to new PCR tubes and incubated. After GEM incubation, the mix was cleaned up using DynaBeads (Thermo Fisher Scientific) and SPRIselect beads (Beckman Coulter). Post-GEM quality control (QC) was performed using an Agilent Bioanalyzer High Sensitivity DNA chip (Agilent) to determine yield and fragment size. DNA-seq libraries were then constructed. Following library construction, Hybridization-based enrichment was performed on DNA-seq libraries using a custom SureSelect bait panel (Aligent) designed to target *AR* and other defined genomic regions (Supplementary Data S1). Post-capture DNA-seq libraries were pooled and sequenced on an Illumina HiSeq 2500 using 2 × 150 bp settings. Raw data are deposited in the NCBI database of genotypes and phenotypes (dbGaP) under accession phs003343.v1.p1.

### Linked-read DNA-seq data analysis

The 10× Genomics Long Ranger pipeline was used for alignment to the hg19 human reference sequence. Targeted DNA-seq samples were analyzed using the ‘longranger targeted’ mode and WGS samples were analyzed with the ‘targeted wgs’ mode. The Long Ranger pipeline provides SV calls using the barcode-aware Lariat algorithm (26) and SNV calling with either freebayes (27) or mutect2 (28). We restricted SNV calls to those filtered as PASS and annotated as Oncogenic or Likely Oncogenic in the OncoKB database (29). NAIBR was used as an additional SV calling tool (30). In NAIBR calling, three different -d parameter values (1000, 5000 and 10 000) were used and at least 5 split molecules of support were required for a call to be included in the final NAIBR SV call set. Barcode visualizations were created using code that was partially derived from the gemtools software package (31). *AR* copy CNV estimates were made as previously described (9,13). CNV estimates of other genes covered by the targeted bait panel were made as previously described (32).

### Whole genome short-read DNA-seq data analysis

Datasets describing CNVs, SVs and gene expression in whole-genome short-read DNA-seq and RNA-seq datasets from 101 CRPC biopsies have been described (8,9).

### Genomic PCR

PCR primers were designed to amplify specific *AR* structural variant breakpoints (Supplementary Table S1). Genomic DNA from PDX tissue was subjected to whole genome amplification (WGA) using the REPLI-g Amplification kit (Qiagen). WGA DNA or DNA that was isolated directly from PDX tissue was used in PCR using the AccuStart II PCR SuperMix (Quanta Bioscience).

### Optical genome mapping

LuCaP 105 and 105CR tumors sized approximately 1000 mm^3^ were resected from mice, minced into 100–200 mm^3^ bits, and flash frozen. Tumor bits were shipped on dry ice to Bionano Genomics (San Diego, CA) and used in the Bionano SP Tissue and Tumor DNA Isolation protocol for 400× Human Genome Sample Analysis. Tumor cells were enzymatically digested and lysed, and ultra-high molecular weight genomic DNA was bound by a paramagnetic disk in isopropanol. DNA was washed, eluted and homogenized in buffer, and quantified. A 750 ng aliquot of ultra-high molecular weight DNA was as input for the Bionano Prep Direct Label and Stain (DLS) procedure, wherein the DLE-1 enzyme conjugated fluorescent labels at the occurrence of a 6 bp motif. Excess labels were removed, and the DNA backbone was counterstained overnight. Labeled DNA was transferred to flowcells of a Saphyr Chip, then linearized and scanned with the Saphyr System. Images were digitized into whole-genome molecule and label information in real time, and collected to a minimum genome-wide target coverage of 400×.

The dataset was assessed for quality control based on molecule size, labeling efficiency, and map rate and coverage of the hg19 reference. Data analysis was performed with the Bionano Solve Rare Variant Analysis (v 3.7), which performs alignment of individual molecules to this reference. Molecule clusters capturing putative structural variants (SVs) were identified, assembled locally into consensus maps, and realigned to the reference to generate refined and annotated SV calls. Using a separate algorithm based on depth of coverage, a whole genome copy number was also calculated. The copy number profile was analyzed for segments representing regions of copy number gain or loss. SV and copy number variant (CNV) calls were then presented and dynamically visualized in Bionano Access Software (v 1.7).

### Fluorescence *in situ* hybridization (FISH)

VCaP and LuCaP 105CR cells were harvested using 0.05% Trypsin/EDTA following overnight arrest in medium containing 50 ng/ml colcemid. Trypsinized cells were treated with 0.75 M KCl, fixed with 3:1 methanol:acetic acid, and spread onto glass slides. Frozen PDX and CRPC tumor tissue was partially thawed and touched to glass slides. BAC DNA probes encompassing the AR locus (RP11-807F19 and RP11-963N10) were labeled by nick translation reaction using a Nick Translation Kit (Abbott Molecular) and red - 500 dUTP (Enzo Life Science). BAC probes encompassing 16p11.2 (RP11-114A14 and RP11-279M12) were labeled using Green – 500 dUTP (Enzo Life Science). The labeled DNA was precipitated in COT-1 DNA, salmon sperm DNA, sodium acetate and 95% ethanol, dried and resuspended in 50% formamide hybridization buffer, and mixed with commercial probes recognizing the centromeric region of chromosome X (Abbott Molecular). The probe/hybridization buffer mix was denatured, probe was applied to glass slides, and slides were hybridized for 48 h at 37°C in a humidified chamber. After hybridization, the FISH slides were washed in a 2× SSC solution at 72°C for 15–30 s, and counterstained with DAPI stain. Fluorescent signals were visualized on an Olympus BX61 microscope workstation (Applied Spectral Imaging, Vista, CA) with DAPI and FITC filter sets. FISH images were captured using an interferometer-based CCD cooled camera (ASI) and FISHView ASI software.

### Western blot analysis

LuCaP 105 and LuCaP 105CR tumor bits were pulverized under liquid nitrogen, lysed in 1× Laemmli buffer, sheared through a 16-gauge insulin syringe, and boiled. Aliquots of lysates containing 30 μg protein mass were subjected to western blot as described (33) using primary antibodies specific for AR diluted 1:2000 (SP107, Abcam), AR-V7 diluted 1:1000 (AG10008, Precision antibody), AR-V9 diluted 1:1000 (generated under collaboration with RevMab), and tubulin diluted 1:4000 (sc-5286, Santa Cruz).

### RT-PCR

Total RNA (1 μg) was used for cDNA synthesis using cDNA qScript SuperMix (Quanta Biosciences). cDNA (1 ul) was used for quantitative PCR detection of splice junctions specific to full-length AR, AR-V7 and AR-V9 as described (34).

### AR-targeted long-read RNA-seq

Total RNA (50 ng) was used for AR 3’RACE reactions using a 5’/3’RACE kit, second generation (Roche) as described (34). The final AR 3’RACE products were purified using a QIAquick PCR Purification Kit (Qiagen) and submitted to the University of Minnesota Genomics Center where they were converted to barcoded SMRTbell libraries using the PacBio Barcoded Adapters for Multiplex SMRT Sequencing protocol as per the manufacturer's recommendations (Pacific Biosciences). Barcoded SMRTbell libraries were pooled and prepared for diffusion loading on a Pacific Biosciences Sequel and sequenced using Sequel 3.0 chemistry. Circular consensus reads were trimmed, aligned, collapsed into transcripts and visualized as described (9).

## RESULTS

### Benchmarking *AR-*targeted linked-read DNA-seq

**Figure 1.**
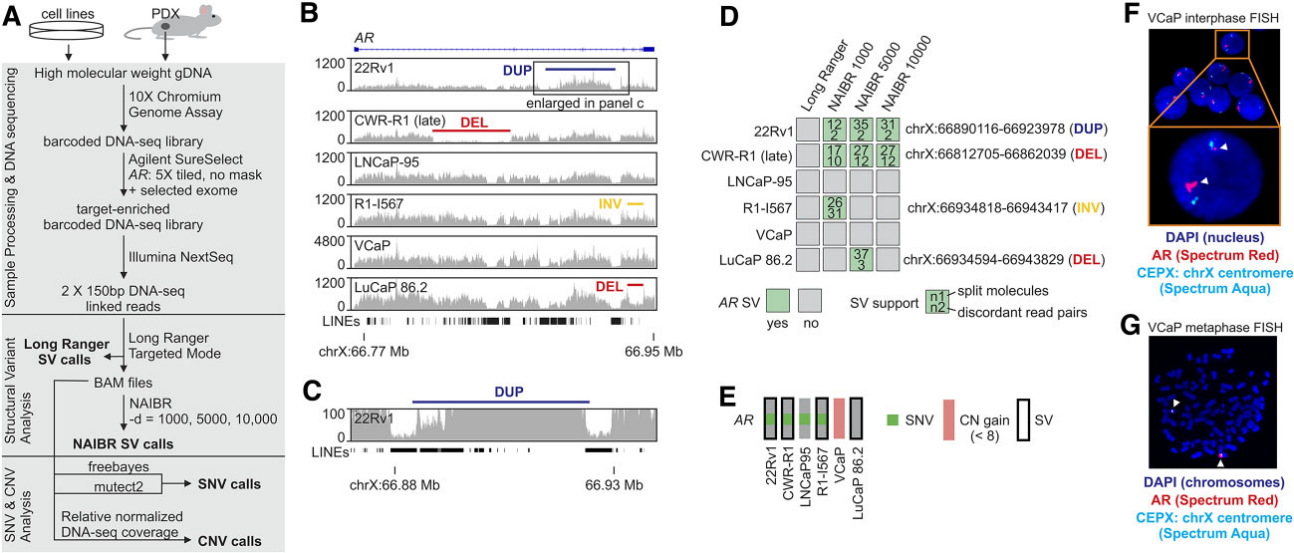
Benchmarking *AR*-targeted linked-read DNA-seq for *AR* genotyping. (**A**) Workflow for targeted linked-read DNA-seq detection of *AR* gene rearrangements (or structural variants, SVs), and single-nucleotide variants (SNVs) and copy number variants (CNVs) in *AR* and other targeted genes. (**B**) DNA-seq read coverage across the *AR* locus in PC cell lines and a PC PDX model with known duplications (DUP), deletions (DEL), and inversions (INV) impacting *AR* structure. Long interspersed nuclear elements (LINEs) are shown. (**C**) Non-zero DNA-seq read coverage in the LINEs harboring DUP breakpoints in 22Rv1 cells. (**D**) AR gene rearrangements (*AR*-SVs) identified in linked-read DNA-seq data using Long Ranger and NAIBR with 3 different d-parameters (1000, 5000 and 10 000). (**E**) Oncoprint summarizing *AR* single nucleotide variants (SNVs), copy number (CN) gain and structural variants (SVs) detected in PC cell lines using *AR*-targeted linked-read DNA-seq. (**F**) Fluorescence *in situ* hybridization (FISH) of VCaP cells in interphase using fluorescence probes targeting AR (red) and chromosome X centromere (aqua). (**G**) FISH of VCaP cells in metaphase as in (F). White arrowheads denote *AR* FISH signal.

We developed a targeted linked-read DNA-seq workflow to enable discovery and DNA molecule phasing of *AR* gene alterations in CRPC (Figure 1A). Linked-read DNA-seq libraries were subjected to hybridization-based enrichment of the *AR* locus and exons of genes known to be altered in CRPC (Supplementary Data S1) followed by Illumina paired-end DNA-seq. Barcode-linked DNA-seq reads were mapped to the human reference genome using barcode-aware Long Ranger software provided by 10× Genomics. Structural variant calling was performed using Long Ranger and NAIBR (30).

We benchmarked this workflow using a panel of three PC cell lines (22Rv1, late-passage CWR-R1, R1-I567) and one PDX (LuCaP 86.2) that are known to harbor *AR* gene rearrangements (15,20,35) and two PC cell lines that lack *AR* gene rearrangements (LNCaP-95 and VCaP). This workflow provided an average 68–524X coverage depth of all targeted regions in these samples, and a median 15–148 linked reads per high molecular weight DNA molecule, yielding phase blocks with median sizes of 17 245–44 955 kb (Supplementary Data S2). We observed high depth of coverage across the *AR* gene in these samples, with lower but non-zero coverage across repetitive DNA elements such as long interspersed nuclear elements (LINEs) (Figures 1B, C). Non-zero coverage in LINEs is important since they often contain *AR* rearrangement breakpoints, as exemplified by an intra-*AR* tandem duplication occurring in 22Rv1 cells (Figures 1B, C). Higher DNA-seq coverage across the *AR* locus was noted in VCaP relative to other models, which reflects *AR* amplification in this cell line (Figure 1B).

We tested the accuracy of two algorithms for detecting known *AR* gene rearrangements in mapped DNA-seq data from these samples. Long Ranger identifies distant loci containing DNA-seq reads linked by the same barcode, indicating structural variation. NAIBR identifies DNA structural variants from split molecules, which are structurally-altered segments of DNA built *in silico* from DNA-seq reads linked by the same barcode. Three NAIBR d-parameter settings were tested, which defines a maximum distance allowed between linked reads. Long Ranger did not detect any known *AR* gene rearrangements, whereas NAIBR identified split molecules and discordant read pairs that correctly resolved the known *AR* gene rearrangements when multiple d-parameter settings were applied (Figure 1D, Supplementary Data S3).

Additionally, algorithms for calling single nucleotide variants (SNV) and copy number variants (CNV) correctly identified a known *AR* H874Y mutation in 22Rv1, CWR-R1, and R1-I567 cells, a known *AR* T878A mutation in LNCaP95 cells, and *AR* amplification in VCaP cells (Figure 1E, Supplementary Data S4 and S5). Fluorescence *in situ* hybridization (FISH) for *AR* and the chromosome X centromere revealed that VCaP cells had 2 copies of chromosome X, one of which contained a single *AR* puncta and the other containing a homogeneously staining region (HSR) of focal *AR* amplification (Figures 1F&G). These benchmarking data indicate that targeted linked-read DNA-seq can provide accurate genotyping of *AR* in PC cells and tissues.

### *AR-*targeted linked-read DNA-seq reveals heterogeneous *AR* gene rearrangements

**Figure 2.**
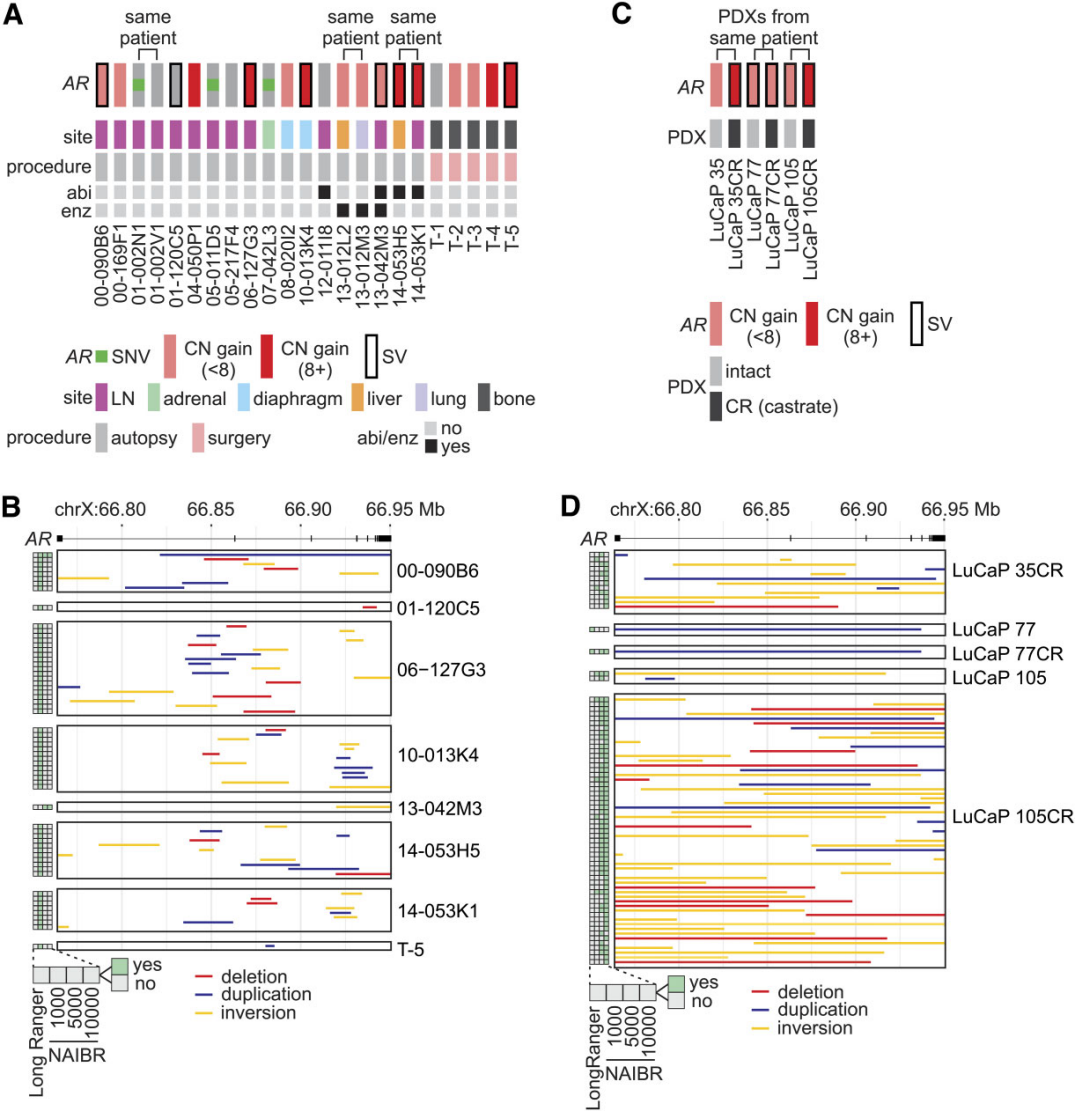
*AR-*targeted linked-read DNA-seq analysis of CRPC tumors. (**A**) Oncoprint summarizing *AR* single nucleotide variants (SNVs), copy number CN) gain, and structural variants (SVs) detected in 23 CRPC tumors from 20 patients treated by androgen depletion. Patients that were also treated with abiraterone (abi) and/or enzalutamide (enz) are indicated. LN, lymph node. (**B**) *AR* gene rearrangements identified in CRPC tumors from A using Long Ranger and NAIBR with 3 different d-parameters (1000, 5000 and 10 000). (**C**) Oncoprint summarizing *AR* copy number (CN) gain and SVs detected in six patient-derived xenograft (PDX) tumors grown in intact or castrate (CR) mice. (**D**) *AR* gene rearrangements identified in PDX tumors from C using Long Ranger and NAIBR as in B.

We used *AR*-targeted linked-read DNA-seq to analyze a cohort of 23 CRPC metastases from surgery or autopsy of 20 patients that had all received androgen depletion therapy, but had variable treatment exposures to abiraterone and enzalutamide (Figure 2A, Supplementary Data S2 and S6–S8). In this cohort, *AR* amplification occurred in 15/23 samples (65%), *AR* SNVs in 3/23 samples (13%), and *AR* gene rearrangements in 8/23 samples (35%). As observed in previous studies, *AR* gene rearrangements mainly occurred in samples that harbored *AR* amplification (9,13). In 5 of the 8 samples with *AR* gene rearrangements, there was evidence for these tumors harboring more than one *AR* gene rearrangement (Figure 2B). *AR* gene rearrangements were heterogeneous between patients and within patients. For instance, 14-053H5 and 14-053K1 are anatomically distinct metastases from the same patient that displayed mutually exclusive sets of *AR* gene rearrangements. This is consistent with previous studies showing that *AR* gene alterations develop under the selective pressure of AR-targeted therapy, which is standard-of-care for disease that has already metastasized (9,13,36).

To understand the evolution of *AR* gene alterations during therapy, we analyzed three PDX models developed from CRPC metastases that were either propagated in intact mice, or serially propagated in castrated (CR) mice to model androgen depletion therapy (Figure 2C, Supplementary Data S2 and S6–S8). LuCaP 35 tumors grown in intact or castrated mice displayed *AR* amplification, with accumulation of *AR* copy number and 12 *AR* gene rearrangements in LuCaP 35CR (Figures 2C, D). LuCaP 77 tumors grown in intact or castrated mice displayed *AR* amplification and one *AR* gene rearrangement that we confirmed using PCR and Sanger sequencing (Figures 2C, D, Supplementary Figure S1). LuCaP 105 tumors grown in intact or castrated mice displayed *AR* amplification as well as multiple *AR* gene rearrangements, with a much higher *AR* copy number and burden of *AR* gene rearrangements in LuCaP 105CR (Figures 2C, D). PCR and Sanger sequencing confirmed that an inversion observed in LuCaP105 was retained in LuCaP 105CR (Supplementary Figure S2A–D), and confirmed that several *AR* gene rearrangements detected in LuCaP 105CR were undetectable in LuCaP 105, indicating they emerged under castration pressure (Supplementary Figure S2E–J). Overall, these findings from paired PDX tissues indicate that *AR* gene rearrangements accumulate under the stress of androgen depletion.

### Whole genome linked-read DNA-seq reveals evolution of AR gene rearrangements

**Figure 3.**
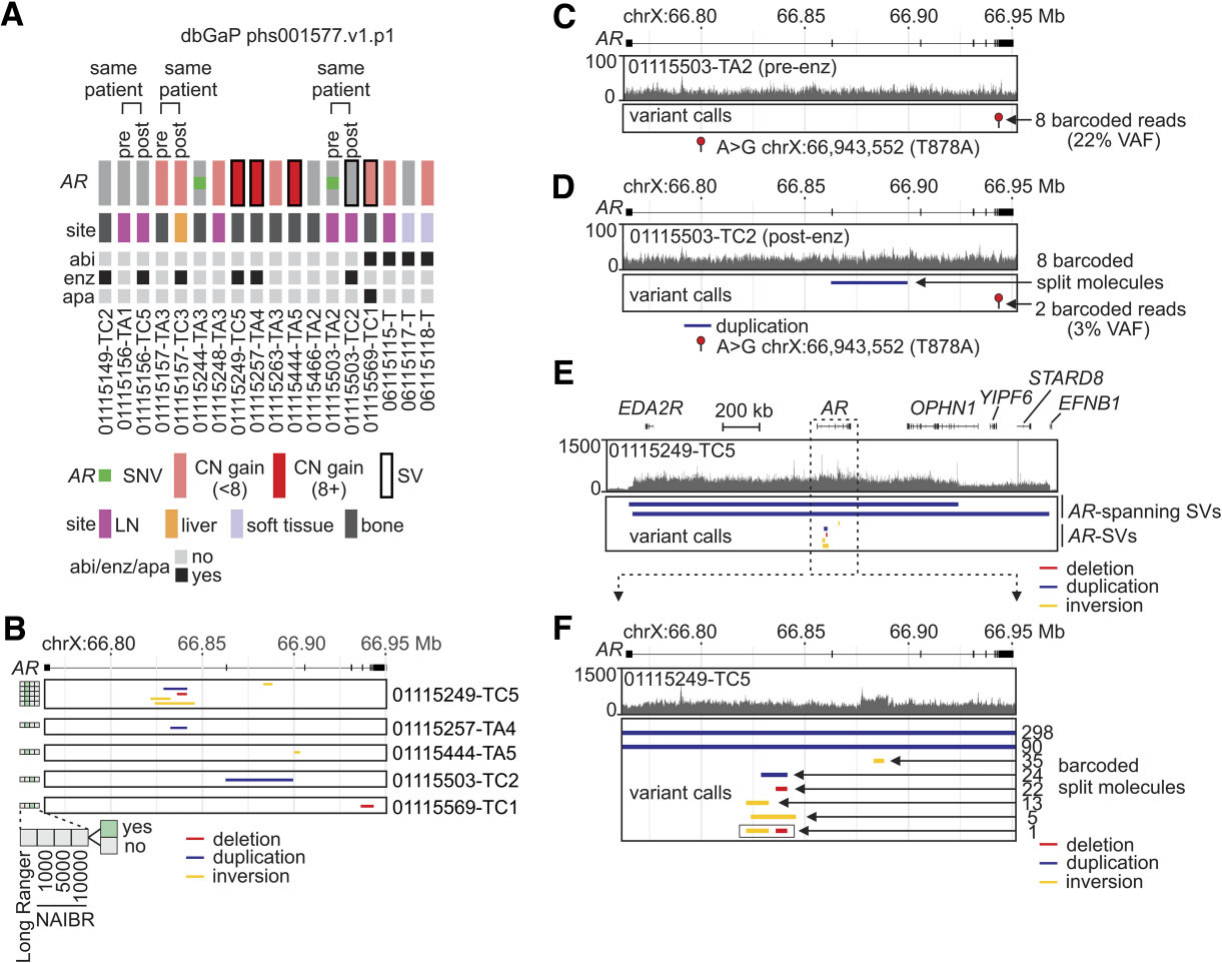
*AR* genotyping in whole-genome linked-read DNA-seq data. (**A**) Oncoprint summarizing *AR* single nucleotide variants (SNVs), copy number (CN) gain and structural variants (SVs) in 18 CRPC tumors from 15 patients treated by androgen depletion. Patients that were also treated with abiraterone (abi), enzalutamide (enz), and/or apalutamide (apa) are indicated. LN, lymph node. (**B**) *AR* gene rearrangements identified in CRPC tumors from (A). (**C**) DNA-seq read coverage and read support for an *AR* T878A mutation in the pre-treatment CRPC sample TA2 from patient 01115503. (**D**) DNA-seq read coverage, NAIBR split molecule support for a duplication, and DNA-seq read support for an *AR* T878A mutation in the enzalutamide post-treament sample TC2 from patient 01115503. (**E**) DNA-seq read coverage and NAIBR split molecule support for SVs within and flanking *AR* in a CRPC tumor (TC5) from patient 01115249. (**F**) Zoomed in view of panel (E), showing the number of barcoded split molecules identified by NAIBR supporting *AR* gene rearrangements. One split molecule supported phasing of a duplication and an inversion.

We analyzed whole genome linked-read DNA-seq data from a separate cohort of 18 CRPC metastases from 15 patients that had all received androgen depletion therapy, which included pre- and post-treatment samples from three patients treated with enzalutamide (17). In this cohort, the frequencies of *AR* amplification (10/18 or 55% of samples), *AR* SNVs (2/18 or 11% of samples) and *AR* gene rearrangements (5/18 or 27% of samples) were similar to the frequencies observed in the cohort we analyzed by targeted linked-read DNA-seq (Figure 3A, Supplementary Data S9–S11). Further, all *AR* gene rearrangements were heterogeneous between patients, but were fewer in number than observed by targeted linked-read DNA-seq, which is likely due to the shallower (average 21–45× coverage (17)) sequencing depth (Figure 3B). Combining the data from all 41 samples analyzed by targeted and whole genome linked-read DNA-seq showed that *AR* gene rearrangements were more likely to occur in tumors harboring eight or more *AR* gene copies than in tumors harboring less than 8 *AR* gene copies (8/10 versus 5/31, *P* = 0.0005, Fisher's exact test). Integrative analysis of a separate whole genome DNA-seq dataset (non-linked read) from 101 CRPC metastases (8,9) revealed a similar enrichment of *AR* gene rearrangements in tumors harboring eight or more AR gene copies (11/19 versus 12/82, *P* = 0.0002, Fisher's exact test), indicating this relationship is not an artifact of linked-read DNA-seq (Supplementary Figure S3).

In the cohort analyzed by whole genome linked-read DNA-seq, patient 01115503 displayed evolution of *AR* status during treatment with enzalutamide, evidenced by presence of an *AR* T878A mutation prior to treatment and emergence of an *AR* gene rearrangement (tandem duplication) after treatment (Figures 3C, D). Manual inspection of DNA-seq reads identified 2 reads that supported the *AR* T878A mutation in the post-treatment sample, indicating this clone was still present but had dropped to a level that was below the 10% variant allele threshold we used (Supplementary Figure S4). There were eight split molecules identified by NAIBR that supported the emergent tandem duplication in the enzalutamide post-treatment sample, but none that supported this tandem duplication in the pre-treatment sample. By comparing the barcodes linking the DNA-seq reads in these eight split molecules with the barcodes linking the two DNA-seq reads supporting AR T878A, we observed zero overlap. The differences observed pre- and post-treatment could reflect tumor heterogeneity and biopsy sampling bias, or reflect clonal evolution wherein tumor cells harboring the intra-*AR* tandem duplication emerged during therapy with enzalutamide, and tumor cells harboring the *AR* T878A mutation became less abundant.

One sample from the cohort analyzed by whole genome linked-read DNA-seq (01115249-TC5) was found to harbor multiple *AR* gene rearrangements. We applied this strategy of investigating barcode overlap to understand the relationships between these multiple *AR* gene rearrangement observed in sample 01115249-TC5. Barcode analysis of split molecules supporting two large tandem duplications flanking the *AR* gene indicated they were distinct from each other as well as split molecules supporting each of the five *AR* gene rearrangements in this sample (Figures 3E, F). Barcode analysis of the split molecules supporting each of the five *AR* gene rearrangements indicated they were largely independent, with the exception of one split molecule that supported both an inversion and a deletion (Figure 3F). This suggested that the inversion and the deletion were both contained on the same DNA molecule, and raised the possibility that co-occurrence of multiple *AR* gene rearrangements in a tumor reflects the presence of complex, multiply-rearranged *AR* gene structures.

### Multiply-rearranged *AR* gene structures are associated with high-level *AR* amplification

**Figure 4.**
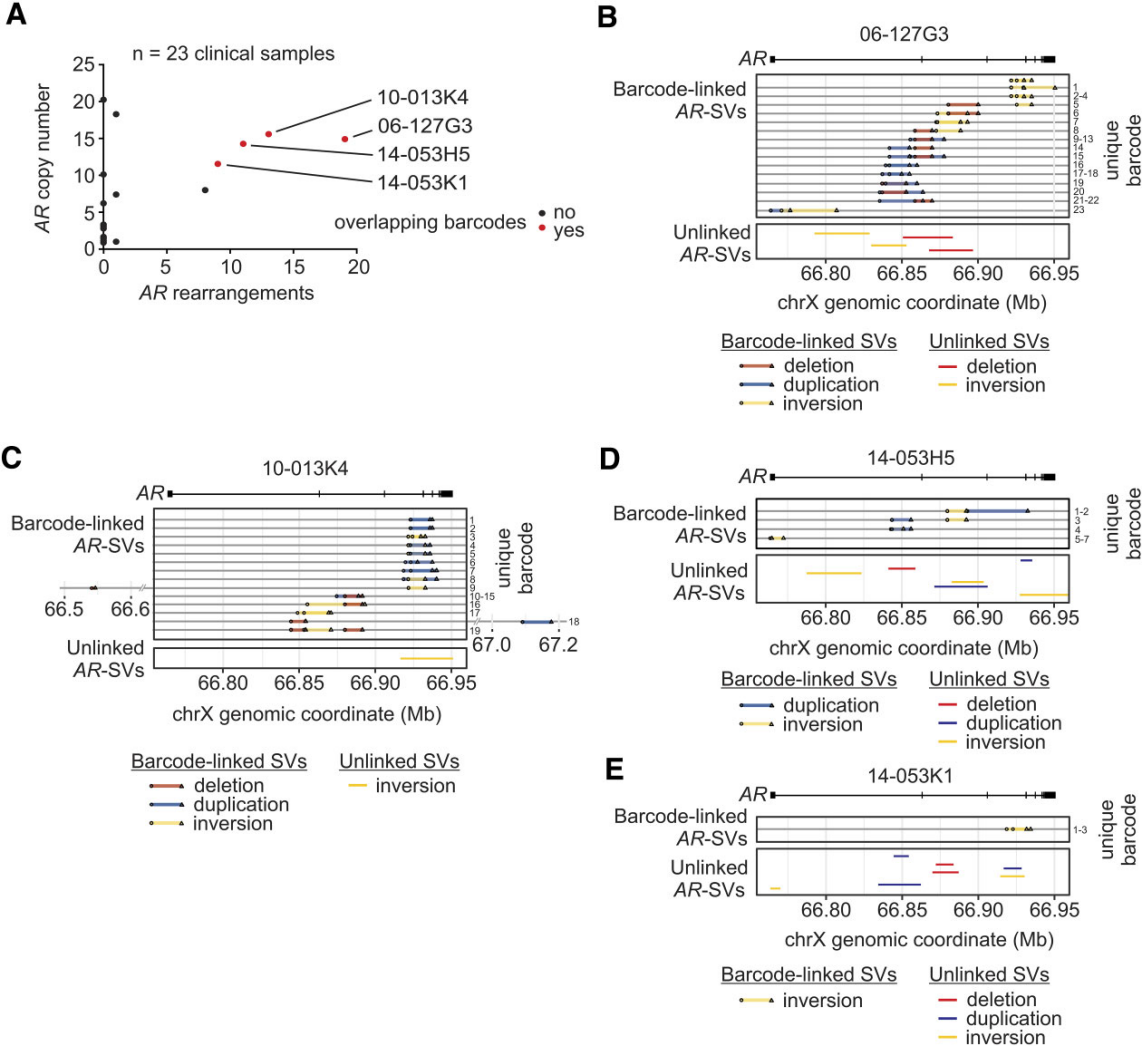
Multiply-rearranged *AR* gene structures in CRPC tumors. (**A**) *AR* copy number and *AR* gene rearrangements identified in 23 CRPC tumors from 20 patients analyzed by *AR*-targeted linked-read DNA-seq. Red color denotes tumors with a NAIBR split molecule supporting an *AR* gene rearrangement that shared the same barcode with at least one other NAIBR split molecule supporting a separate *AR* gene rearrangement. (**B–E**) Four CRPC tumors displaying barcode overlap between NAIBR split molecules supporting different *AR* gene structural variants (SVs) (barcode-linked AR-SVs). Unlinked *AR*-SVs supported by NAIBR split molecules that did not share barcode overlap with other NAIBR split molecules are shown.

To pursue this further, we assessed barcode overlap in deep *AR*-targeted linked-read DNA-seq data from the cohort of 23 CRPC specimens. In this dataset, 4 CRPC specimens (17%) had split molecules with overlapping barcodes, indicating complex *AR* gene rearrangements. Noteworthy, these 4 CRPC specimens (10-013K4, 06-127G3, 14-053H2 and 14-053K1) ranked among the highest in the number of *AR* gene copies and burden of *AR* gene rearrangements (Figure 4A).

Of the 19 *AR* gene rearrangements identified in CRPC tumor 06-127G3, 15 had split molecules with at least one barcode that shared overlap with a separate split molecule (Figure 4B). Notably, most of these *AR* gene rearrangements were phased with at least two other *AR* gene rearrangements. For instance, a deletion in this sample was phased with an inversion (barcode 8), and several duplications (barcodes 9–15 and 21–22). A similar pattern was observed in CRPC specimen 10-013K4 wherein barcodes 1–9 connected a network of phased duplications and inversions at the 3’ end of the *AR* gene and barcodes 10-19 connected a network of phased inversions, deletions, and duplications in the middle of the *AR* gene (Figure 4C). CRPC tumors 14-053H5 and 14-053K1, which were obtained from the same patient, each displayed unique patterns of phased and unlinked *AR* gene rearrangements (Figures 4D, E).

Across all 41 CRPC specimens analyzed by targeted or whole genome linked-read DNA-seq, phased *AR* gene rearrangements only occurred in tumors that harbored 8 or more *AR* gene copies (5/10 versus 0/31, *P* = 0.0003, Fisher's exact test). These results indicate an association between very high *AR* copy number and complex, multiply-rearranged *AR* gene structures.

### Multiply-rearranged *AR* gene structures emerge with castration pressure in PDXs

**Figure 5.**
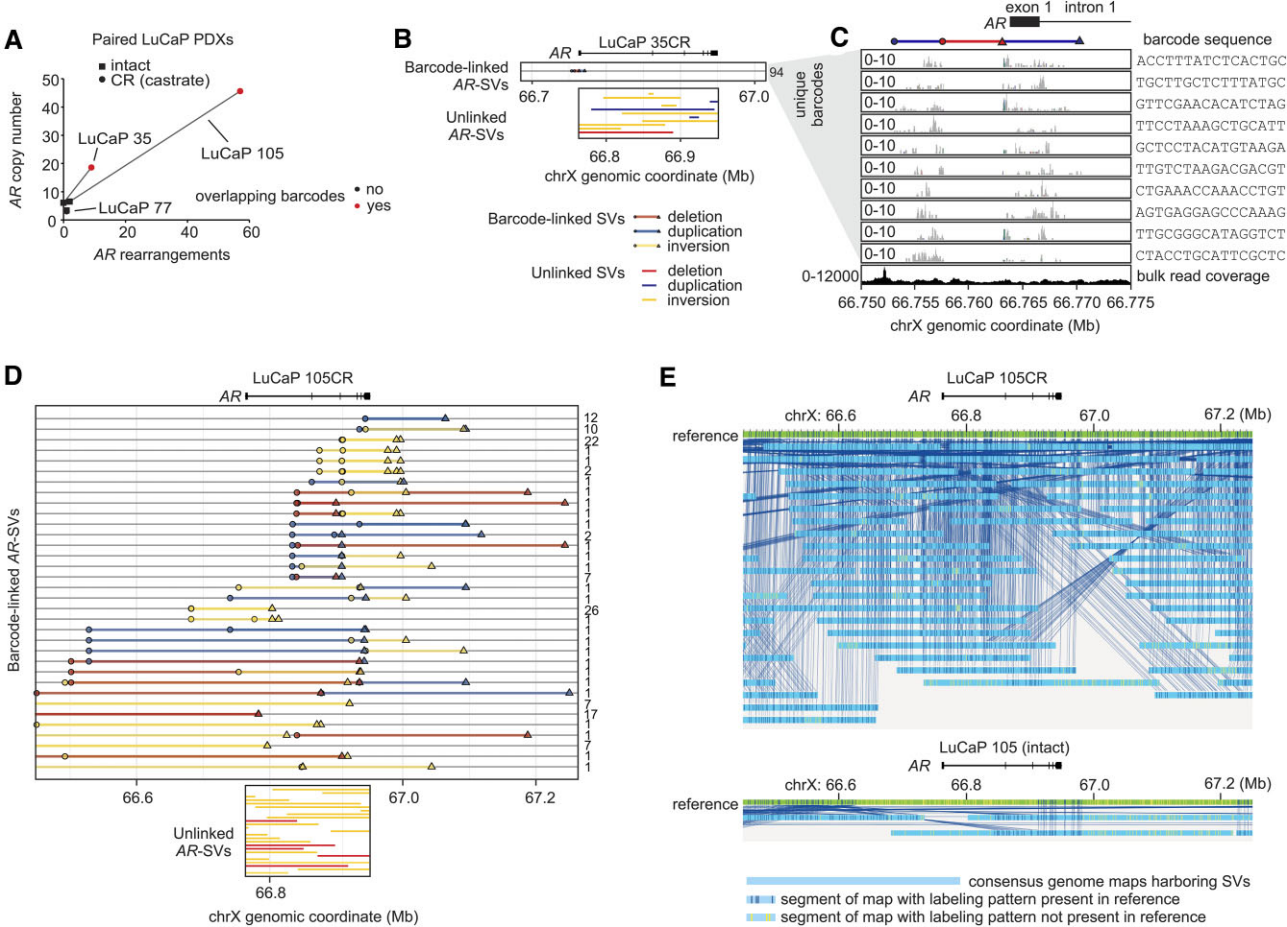
Emergence of multiply-rearranged *AR* gene structures in CRPC PDXs. (**A**) *AR* copy number and *AR* gene rearrangements identified in 6 patient derived xenograft (PDX) tumors by *AR*-targeted linked-read DNA-seq. Red color denotes PDX tumors with a NAIBR split molecule supporting an *AR* gene rearrangement that shared the same barcode with at least one other NAIBR split molecule supporting a separate *AR* gene rearrangement. (**B**) Barcode overlap between NAIBR split molecules supporting a deletion and a duplication in a LuCaP 35CR tumor. Unlinked *AR*-SVs supported by NAIBR split molecules that did not share barcode overlap with other NAIBR split molecules are shown. (**C**) Read coverage of DNA-seq reads containing 10 barcodes from 10 different split molecules supporting a duplication in LuCaP 35CR tumors. Bulk DNA-seq coverage is shown at the bottom. (**D**) Barcode overlap between NAIBR split molecules supporting *AR*-SVs in a LuCaP 105CR tumor. Unlinked *AR*-SVs supported by NAIBR split molecules that did not share barcode overlap with other NAIBR split molecules are shown. (**E**) Consensus genome maps harboring AR structural variants (SVs) generated from optical genome mapping of LuCaP 105CR (top) and LuCaP 105 (bottom) tumors.

To investigate the basis for this association between very high *AR* copy number and complex multiply-rearranged *AR* gene structures, we focused on LuCaP 35 and LuCaP 105 PDX models, which displayed accumulation of *AR* gene copies and *AR* gene rearrangements under castration pressure (Figures 2C, D). In both LuCaP 35CR and LuCaP 105CR tumors grown in castrated mice, split molecules shared barcode overlap with other split molecules, indicating the emergence of complex, multiply-rearranged *AR* gene structures (Figure 5A).

In LuCaP 35CR, a duplication with a breakpoint occurring in the 5’ end of the *AR* gene was phased with a deletion occurring upstream of *AR* (Figures 5B). We manually inspected coverage of DNA-seq reads that comprised the split molecules containing the duplication and were linked by 10 of these overlapping barcodes. This confirmed a lack of coverage across the deleted region in these split molecules, despite coverage of this region in the bulk DNA-seq data (Figure 5C). In LuCaP 105CR, the majority of the *AR* gene rearrangements were phased with at least one additional *AR* gene rearrangement via shared barcodes, indicating a complex collection of multiply-rearranged *AR* gene structures (Figure 5D).

**Figure 6.**
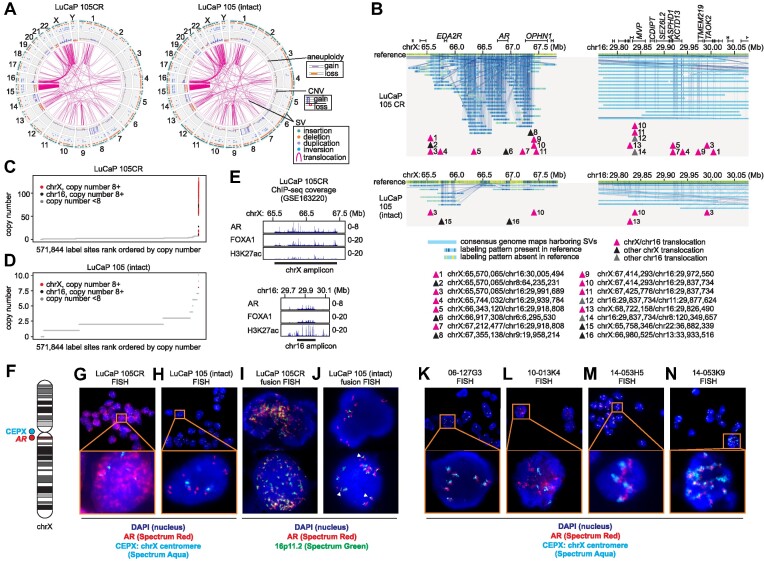
*AR* structural complexity is associated with *AR* ecDNA. (**A**) Circos plots generated from optical genome mapping of LuCaP 105CR and LuCaP 105 tumors, showing copy number variants (CNVs) and structural variants (SVs). (**B**) Consensus genome maps from LuCaP 105CR (top) and LuCaP 105 (bottom) tumors, with LuCaP 105CR displaying a higher degree of structural variation (SV) on chromosomes X and 16, with multiple translocation breakpoints fusing these regions. (**C**) LuCaP 105CR outlier copy number gain profiles for regions of chromosome X and 16 containing translocations shown in (B). (**D**) LuCaP 105 copy number profiles for comparison with (C). (**E**) Chromatin immunoprecipitation and DNA sequencing (ChIP-seq) signals for AR, FOXA1, and H3K27ac across chromosome X and 16 amplicons in LuCaP 105CR. (**F**) Fluorescence *in situ* hybridization (FISH) strategy using fluorescence probes targeting AR (red) and chromosome X centromere (aqua). (**G, H**) FISH of LuCaP 105 and 105CR tumor cells using probes in (F). (**I, J**) FISH of LuCaP 105 and LuCaP 105CR tumor cells using fluorescence probes targeting AR (red) and 16p11.2 (green). (**K–N**) FISH of CRPC tissues using probes in (F).

To confirm the presence of multiply-rearranged *AR* gene structures emerging in LuCaP 105CR tumor tissue using an orthogonal method, we performed optical genome mapping (37). Ultra-high molecular weight DNA fragments were isolated from LuCaP 105 and 105CR, labeled at specific sequences, optically mapped, and assembled into consensus genome maps. These consensus genome maps confirmed a high burden of *AR* gene rearrangements accumulating in LuCaP 105CR relative to LuCaP 105, with most of the consensus genome maps from LuCaP 105CR displaying multiple rearrangements (Figure 5E). Optical genome mapping also confirmed *AR* amplification in LuCaP 105 and LuCaP 105CR (Figure 6A). The agreement between independent techniques of AR-targeted linked-read DNA-seq and optical genome mapping supports the conclusion that a high number of multiply-rearranged *AR* gene copies emerged under castration pressure in LuCaP 105CR.

### Multiply-rearranged *AR* gene structures in PDX models occur on ecDNA

In linked-read DNA-seq and optical genome mapping datasets from LuCaP 105CR, the region of structural instability extended upstream and downstream of the *AR* gene (Figures 5D, E). Optical genome mapping data also revealed translocations between chromosomes X (chrX) and 16 (chr16) in both LuCaP 105 and LuCaP 105CR (Figure 6A). These translocations localized to a ∼2 Mb region of chrX encompassing *AR, EDA2R, and OPHN1*, and a ∼200 kb region of chr16 encompassing *MMP, CDIPT, SEZ6L2, ASPHD1, KCTD13, TMEM219, and TAOK2* (Figure 6B). When we analyzed copy number at optical mapping label positions on a genome-wide scale, the labels at these two genomic regions displayed outlier copy number values, only in LuCaP 105CR (Figures 6C, D). Outlier copy number of these two genomic regions was also evident from visualization of ChIP-seq data from a published study of LuCaP 105CR tumor tissue (38), signified by high background signal throughout the amplified regions (Figure 6E). Interestingly, these ChIP-seq data also demonstrated a high density of FOXA1 and AR binding, and high density of the active enhancer mark of histone H3 acetylated on lysine 27 (H3K27ac), indicating these amplicons harbored transcriptionally active chromatin.

Based on these data, we hypothesized that *AR*, *AR* flanking regions, and a segment of chr16 were amplified on extrachromosomal double-minutes (more recently referred to as ecDNA (18)) in LuCaP 105, and these ecDNA molecules accumulated to high levels under castration selection in LuCaP 105CR. Using FISH for *AR* and the chrX centromere, we confirmed *AR* ecDNA staining in all nuclei of LuCaP 105CR tumor cells from castrated mice (Figures 6F, G and Supplementary Figure S5A). This AR ecDNA pattern also occurred in most nuclei from LuCaP 105 tumor cells grown in intact mice, but with lower signal intensity (Figure 6H). Using a fusion FISH strategy, we confirmed that many of the *AR* ecDNA molecules in LuCaP 105CR tumor cells had signal that overlapped with signal for the chr16 amplicon region at 16p11.2 in LuCaP 105CR tumor cells (Figure 6I and Supplementary Figure 5B). These overlapping signals were detectable in LuCaP 105 tumor cells grown in intact mice (Figure 6J). FISH for *AR* also yielded an *AR* ecDNA pattern in the majority of nuclei from LuCaP 35 tumor cells grown in intact mice, as well as LuCaP 35CR tumor cells grown in castrated mice (Supplementary Figure S6A). Importantly, this *AR* ecDNA FISH staining pattern was also evident in the clinical CRPC tumors that were identified by linked-read DNA-seq as having very high AR copy number and multiply-rearranged AR gene structures (Figures 6K–N).

To explore genetic associations with *AR* ecDNA, we assessed SNVs and CNVs in additional CRPC driver genes covered by the linked-read DNA-seq assay (Supplementary Data S12 and S13). LuCaP 105CR tumor tissue displayed heterozygous loss of *PTEN* and homozygous loss of *RB1*, and CRPC tumors 14-053H5 and 14-053K1 shared common mutations in *TP53* and *CTNNB1*. LuCaP 35CR tumor tissue and CRPC tumors 06-127 and 10-013K4 lacked known pathogenic SNVs or CNVs. This pattern indicated that *AR* ecDNA was not associated with a common alteration that could be identified from the CRPC driver genes targeted by our linked-read DNA-seq assay. This aligns with previous work showing that *AR* gene rearrangements are not restricted to CRPC tumors with specific genetic alterations, except for a higher rate of occurrence in tumors with *PTEN* loss (9).

### CRPC tumors with *AR* ecDNA express diverse *AR* mRNAs

**Figure 7.**
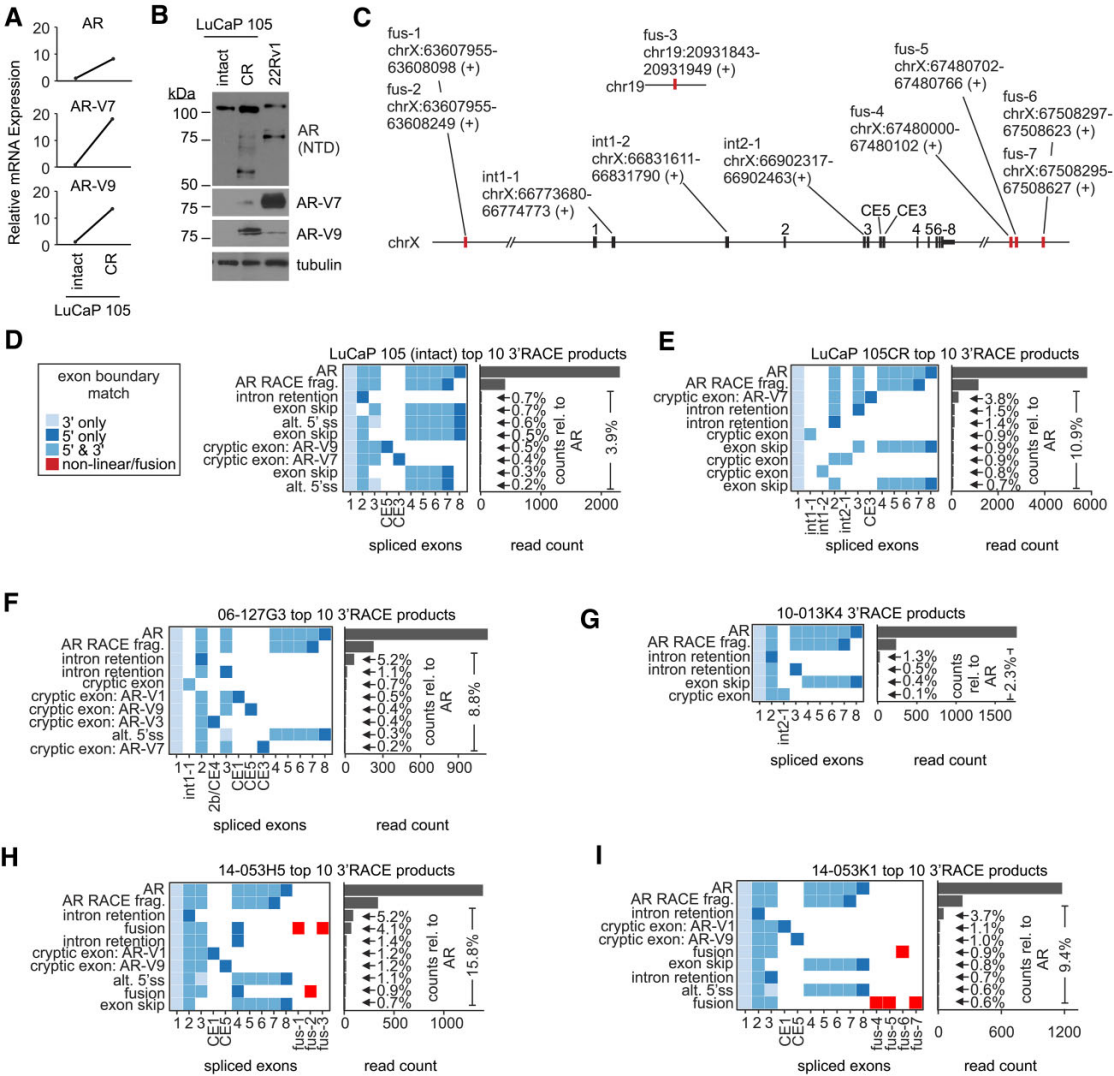
Altered *AR* expression in tumors harboring *AR* ecDNA. (**A**) Levels of indicated AR mRNA species were quantified in LuCaP 105 and LuCaP 105CR tumors by RT-PCR. (**B**) Western blot of lysates from LuCaP 105 and LuCaP 105CR tumors and 22Rv1 PC cells probed with antibodies specific for the AR N-terminal domain (NTD), AR-V7 and AR-V9. Tubulin is a loading control. (**C**) Summary of exons spliced in AR mRNAs isolated from LuCaP 105, LuCaP 105CR and four CRPC tissues. Exons originating outside the AR locus are red. (**D–I**) Exon composition and quantification of the 10 most abundant *AR* transcripts identified by 3’ RACE and PacBio sequencing in mRNA isolated from indicated PDX tissues and CRPC specimens. Individual pixels represent discrete exons contained in individual *AR* transcripts. Pixel colors indicate whether that exon was spliced via annotated splice sites at the 5’ and/or 3’ ends of known exons. Transcripts are annotated based on splicing alteration. Read counts represent the number of single molecule transcripts that matched the indicated splicing pattern. ‘AR RACE frag.’ represents internal priming of full-length *AR* transcripts at an A-rich region in exon 7, which are included as full-length *AR* transcripts for calculation of abundances of alternatively spliced variants shown as % relative to full-length *AR*.

Continuous structural evolution of *AR* ecDNA is likely the mechanistic basis for the high burden and complexity of rearranged *AR* gene structures observed in this subset of CRPC tumors. As expected from the much higher *AR* copy number in LuCaP 105CR vs. LuCaP 105 tumor tissue, the levels of full-length *AR* mRNA and protein were also higher (Figures 7A, B). LuCaP 105CR tumor tissue also displayed emergence of myriad smaller AR protein species with molecular weights ranging from ∼60–80 kDa, at least 2 of which were identified as AR-V7 and AR-V9 (Figures 7A, B). This is potentially significant as these are truncated, constitutively active forms of AR that promote CRPC resistance to endocrine therapies (19,34,39). Emergence of multiple AR protein species in this ∼60–80 kDa size range was also observed in LuCaP 35CR tumor tissue (Supplementary Figure S6B). Previously, we detected AR-V7 and AR-V9 mRNA in LuCaP 35CR tumor tissue using an *AR*-targeted long-read RNA-seq assay (34). Noteworthy, in that previous study we also detected many additional *AR* mRNA transcripts in LuCaP 35CR tumor tissue arising from cryptic exon inclusion, intron retention, and alternative 5’ and/or 3’ splice site usage. If translated, these *AR* mRNA species would produce AR proteins with molecular weights corresponding to those observed in western blots with LuCaP 35CR lysates (Supplementary Figure S6B).

To explore this further, we used *AR*-targeted long-read RNA-seq to characterize *AR* mRNA splicing in the CRPC tumors that displayed a high burden and complexity of *AR* gene structures with *AR* ecDNA FISH staining. This approach identified a diversity of known and novel exons spliced into *AR* mRNAs (Figure 7C). *AR* transcripts arising from alternative splicing events were present at higher cumulative levels relative to full-length *AR* transcripts in LuCaP 105CR versus LuCaP 105 (10.9% versus 3.9%), which included a higher relative ratio of AR-V7 splicing (3.8% vs. 0.4%, Figures 7D&E) as well as splicing of novel cryptic exons located in *AR* introns 1 and 2 (Figure 7E). Multiple known and novel *AR* mRNAs arising from alternative splicing were also detected in the clinical CRPC tumors that displayed *AR* ecDNA FISH staining (Figures 7F–I). The highest levels of cumulative alternative splicing relative to full-length *AR* mRNA occurred in two separate tumors from patient 14-053, where AR-V1 and AR-V9 were detected, as were novel *AR* mRNAs arising from splicing of 3’ fusion exons derived from regions upstream of the *AR* locus, downstream of the *AR* locus, as well as chromosome 19 (Figures 7H and I). Collectively, these data demonstrate that the high burden and complexity of rearranged *AR* gene structures on *AR* ecDNA may provide a growth advantage to CRPC tumors by increasing the levels of AR, as well as broadening the diversity of AR mRNAs and proteins.

## DISCUSSION

*AR* is one of the most frequently rearranged genes in CRPC-stage disease (9). A high proportion of CRPC tumors and ctDNA samples harbor multiple *AR* gene rearrangements concurrent with *AR* amplification (9,10,12). Prior studies investigating *AR* gene rearrangements employed Illumina short-read DNA-seq, which can detect DNA rearrangement breakpoints. However, this technique lacks long-range information necessary to understand whether multiple rearrangement breakpoints detected within the *AR* gene reflects different sets of *AR* gene rearrangements between individual cells, or accumulation of multiple *AR* gene rearrangements within individual cells. To address this, we developed and benchmarked an *AR*-targeted linked-read DNA-seq sequencing strategy to analyze *AR* gene structure at high depth, and also analyzed lower-depth whole-genome linked read DNA-seq data. It should be noted that the 10× Genomics platform used to generate both of these datasets has been discontinued, likely because simpler and lower-cost linked-read DNA-seq methods have been developed (40).

Both targeted and whole-genome linked-read DNA-seq confirmed that the *AR* gene is frequently rearranged in CRPC, with 35% of CRPC tumors analyzed by AR-targeted linked-read DNA-seq and 27% of CRPC tumors analyzed by whole genome linked-read DNA-seq displaying *AR* gene rearrangements. Importantly, using the long-range information provided by the linked-read barcodes, we found that many CRPC tumors harbored phased *AR* gene rearrangements, meaning that individual DNA molecules in CRPC tumors harbored multiply-rearranged *AR* gene structures. This indicates that CRPC harboring a high diversity of *AR* gene rearrangements has less intra-tumor heterogeneity than would be expected from a scenario where different *AR* gene rearrangements occur in different CRPC cells. However, further investigation of *AR* gene structure with long-read DNA-seq technologies will be required for validation and a full characterization of phased *AR* gene rearrangements in CRPC. Our data also confirmed previous studies that showed a high degree of inter-tumor and inter-patient heterogeneity of *AR* gene rearrangements (9,10,13,41). This heterogeneity is a challenge that has limited the clinical utility of identifying specific *AR* gene rearrangements or breakpoints within *AR*.

All CRPC tumors harboring phased, multiply-rearranged *AR* gene structures also displayed very high levels of *AR* gene amplification. This indicated that *AR* copy number and *AR* gene rearrangements accumulate together in CRPC cells. This co-accumulation was confirmed in the LuCaP 35 and LuCaP 105 PDX models of CRPC progression, where *AR* gene copies and multiply-rearranged *AR* gene structures both increased under the pressure of castration therapy. Using optical genome mapping of LuCaP 105 and LuCaP 105CR tumors, we validated co-accumulation of *AR* gene copies and a high burden of complex, multiply rearranged *AR* gene structures, and discovered amplification on ecDNA as a likely underlying mechanism. Using FISH, we confirmed amplification of *AR* on ecDNA in LuCaP 105 and LuCaP 35 tumors growth in intact mice, and accumulation of *AR* ecDNA in LuCaP 105CR and LuCaP 35CR tumors grown in castrated mice. Importantly, although ecDNA is known to occur with higher frequency in PDX models than clinical specimens across multiple cancers (42), we demonstrated this *AR* ecDNA FISH staining pattern in all 4 clinical CRPC tumors harboring multiply-rearranged *AR* gene structures. Conversely, the androgen-dependent VCaP cell line, which harbors *AR* amplification but lacks detectable *AR* gene rearrangements, displayed an *AR* HSR FISH staining pattern. These observations are consistent with a model wherein *AR* HSR and/or ecDNA amplification are early events in CRPC progression, but *AR* ecDNA may accelerate further accumulation and structural evolution of *AR* during subsequent lines of therapy with AR inhibitors.

Extrachromosomal circular DNA (eccDNA) is derived from nuclear chromosomal DNA and has been reported in approximately 50% of human cancers, including the PC cell lines PC-3 and DU145 (18,42,43). Large, megabase size eccDNA carrying oncogenes in cancer cells is referred to as ecDNA (18). For instance, ecDNA is a mechanism for *EGFR* amplification in gliomas (44). A recent meta-analysis of DNA-seq data derived from multiple PC cohorts found that 9% of CRPC samples had exceptionally high *AR* copy number, and proposed ecDNA as an underlying mechanism (45). A limitation of our study is that we did not perform optical genome mapping with the clinical specimens analyzed by linked-read DNA-seq to characterize ecDNA more broadly, mainly due to the high amounts of tissue required as input. However, *AR* ecDNA was supported by the punctate pattern of *AR* staining by FISH. Noteworthy, previous studies employing *AR* FISH with circulating tumor cells (CTCs) from CRPC patients often observed punctate *AR* staining patterns, at frequencies similar to HSR staining patterns (46,47). It will be important for future studies to test whether ecDNA and/or HSR patterns of *AR* FISH staining in CRPC could serve as prognostic or predictive biomarkers.

Mathematical modeling has predicted that ecDNA amplification can increase oncogene copy number and intratumoral heterogeneity more effectively than chromosomal amplification (42). Our finding of multiply-rearranged *AR* gene structures in CRPC tumors with *AR* ecDNA indicates that ecDNA amplification can also be a source of continuous gene structural variation to further increase intratumoral heterogeneity. Previous studies have shown that ecDNA can evolve, but mainly via a mechanism of smaller ecDNAs joining to form larger ecDNAs (43,48–50), which appeared to occur between AR ecDNA and chr16 ecDNA in the LuCaP 105 model. To the best of our knowledge, our study is the first report of intragenic rearrangements of genes captured on ecDNA, which in the case of *AR* can enhance structural diversity. A limitation of our study is that the genomic region targeted for linked-read DNA-seq did not cover an enhancer located 650 kb upstream of *AR (*8,17,51), which limited our ability to interrogate the impact of ecDNA on the structural relationships between the *AR* enhancer and *AR* gene body. In previous studies, *AR* gene rearrangements have been shown to promote expression of diverse AR-V species that lack the AR ligand binding domain (9,13,15,20,35,52). Consistent with this, we found the CRPC tumors that accumulated a diversity of structurally complex *AR* gene copies displayed alterations in mRNA splicing and expression of AR-Vs, including but not limited to AR-V7 and AR-V9. Given that AR-V7 expression in CTCs from CRPC patients portends poor outcomes (53,54), and is strongly associated with *AR* gene copy number in CRPC tissues (13,55), it will be important to determine whether *AR* amplification and/or structural diversification on ecDNA is a mechanism that explains AR-V7 detection in clinical assays.

## Supplementary Material

zcad045_Supplemental_Files

## Data Availability

Raw sequencing data are available in the NCBI database of genotypes and phenotypes (dbGaP) under accession phs003325.v1.p1. Uncropped gels and films are illustrated in Supplementary Figure S7. Any additional data underlying this article will be shared on reasonable request to the corresponding author.
