# Supplementary material for: Co-evolution of AR gene copy number and structural complexity in endocrine therapy resistant prostate cancer
Source: NAR Cancer. 2023 Aug 24;5(3):zcad045. doi: 10.1093/narcan/zcad045 (PMC10448862; doi:10.1093/narcan/zcad045)
Supplement: zcad045_Supplemental_Files [file zcad045_supplemental_files.zip › SupplementaryTableS1&FigsS1-S7.pdf]

Supplementary Table S1

Supplementary Figures S1-S7

Co-evolution of *AR* gene copy number and structural complexity in endocrine therapy resistant prostate cancer

Andrej Zivanovic *et al*.

Supplementary Table S1: Genomic PCR Primers				
**Primer ID**	**SV coordinates (hg19)**	**SV ID**	**PDX**	**Sequence**
LuCaP77 Dup F2	ChrX:66049760-66937135	Duplication	LuCaP77/LuCaP77CR	5'-GAGCAATCCAATAGCAAAAGAAA
LuCaP77 Dup B2	ChrX:66049760-66937135	Duplication	LuCaP77/LuCaP77CR	5' -ATCCACATGCTGAGGGAAAA
105CR Del 1 F2	ChrX:6641796-66783036	Deletion	LuCaP105CR	5'-CGCTACCTGACTTCAAACCC
105CR Del 1 R2	ChrX:6641796-66783036	Deletion	LuCaP105CR	5'-CTGCTCCGAGACACAACTTG
105CR Inv 2 TT F2	ChrX:66681912-66803589	Inversion (tail-to-tail breakpoint)	LuCaP105CR	5'-GGTTGATGACTTCCGGCTTC
105CR Inv 2 TT R2	ChrX:66681912-66803589	Inversion (tail-to-tail breakpoint)	LuCaP105CR	5'-GCAAACAAAGAAGCCTGGGA
105CR Inv 7 HH F1	ChrX:65925574-66798852	Inversion (head-to-head breakpoint)	LuCaP105CR	5'-GCGGCAACATGATTGATCCT
105CR Inv 7 HH R1	ChrX:65925574-66798852	Inversion (head-to-head breakpoint)	LuCaP105CR	5'-GGCAAGGGAGGAAAGGGATA
105CR Inv 8 HH F1	ChrX:66445840-66920001	Inversion (head-to-head breakpoint)	LuCaP105CR	5'-CACAAAGACCTCGAACAGCC
105CR Inv 8 HH R1	ChrX:66445840-66920001	Inversion (head-to-head breakpoint)	LuCaP105CR	5'-AACTTCACGCCACTTTCACC
qG21q21.3 B13 F	N/A (Chr21q21.3 control)	N/A (normal control)	N/A	5'-GAAAGGTCACATTTCTAGCTCGTG
qG21q21.3 B13 R	N/A (Chr21q21.3 control)	N/A (normal control)	N/A	5'-AAGATGCTATACACATTCCCAAAA

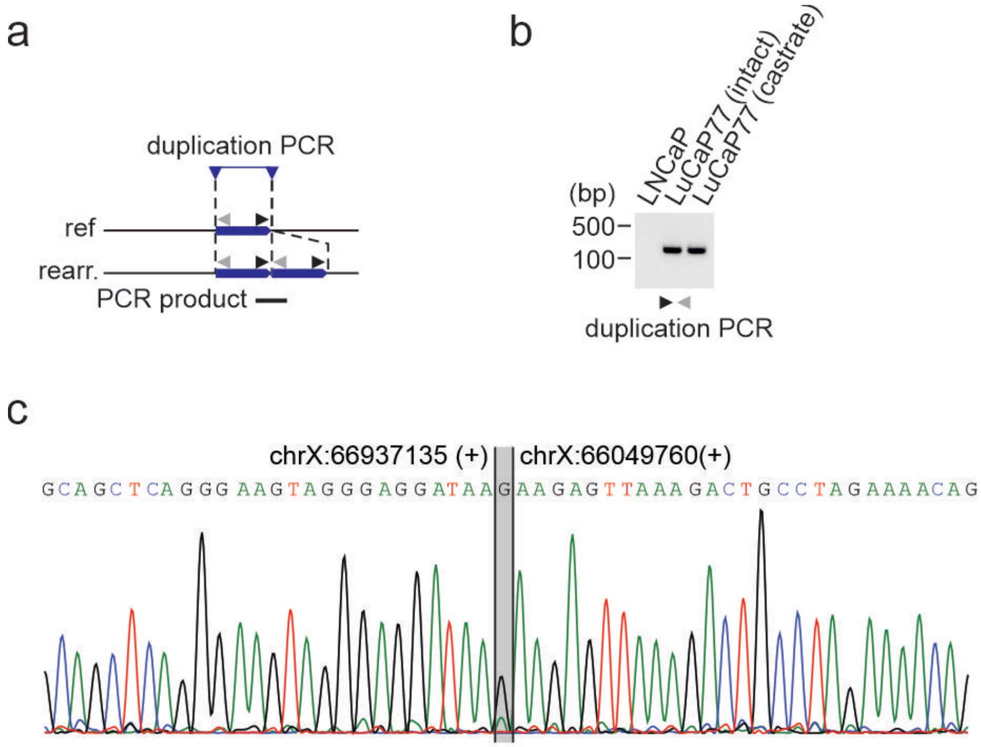

Supplementary Figure S1. PCR validation of a duplication in LuCaP 77 and LuCaP 77CR tumors. A, PCR strategy for detecting tandem duplication breakpoints **B,** Agarose gel electrophoresis of duplication PCR products using DNA isolated from LNCaP cells, a LuCaP 77 tumor, and a LuCaP 77CR tumor. **C,** Sanger sequencing electropherogram of the tandem duplication breakpoint from LuCaP 77CR DNA, using the PCR product from B.

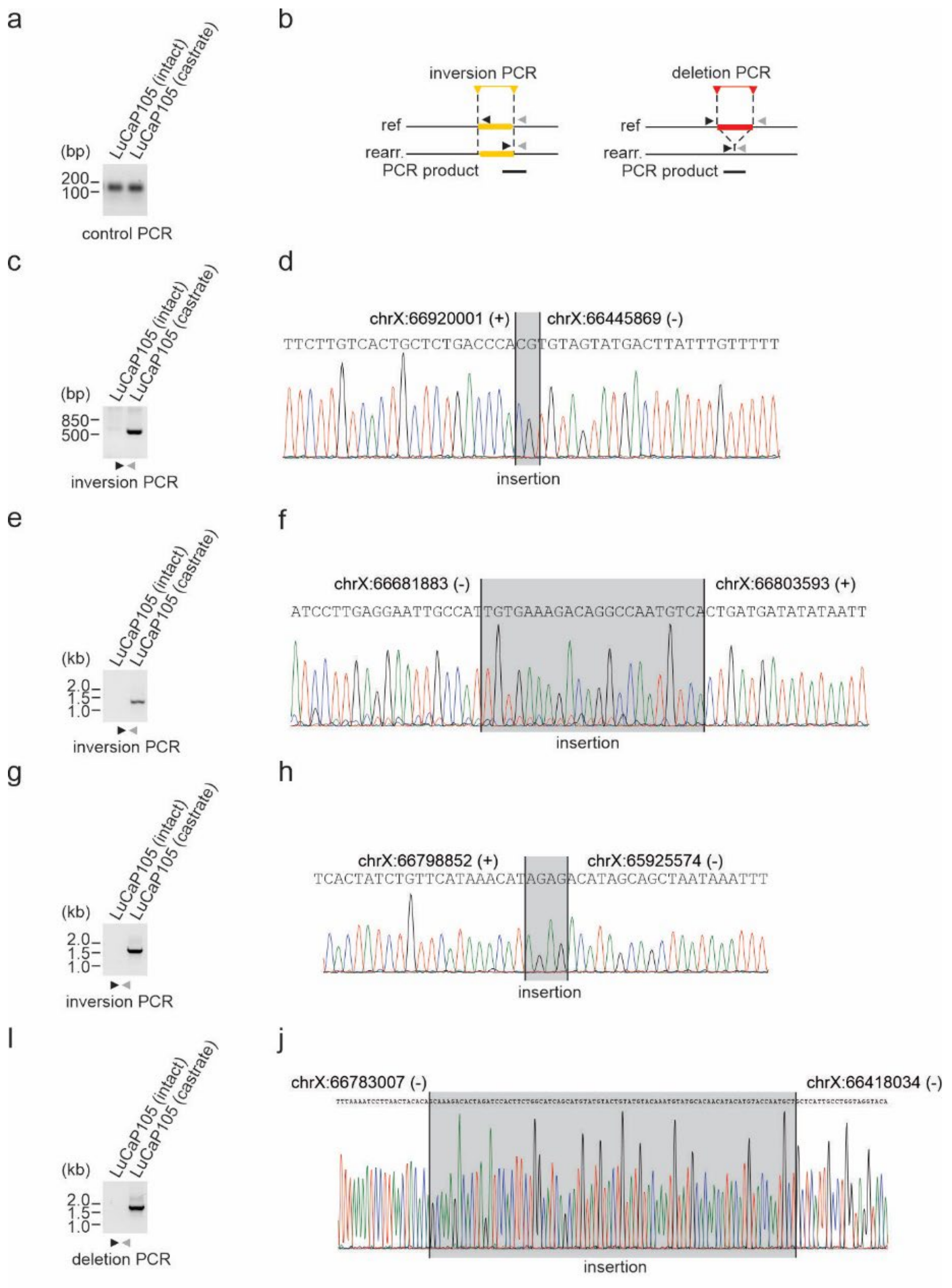

Supplementary Figure S2. PCR validation of *AR* gene rearrangements in LuCaP 105 and LuCaP 105CR tumors. A, Agarose gel electrophoresis of control PCR products using DNA isolated from a LuCaP 105 tumor and a LuCaP 105CR tumor. **B,** PCR strategies for detecting inversion and deletion breakpoints **C,** Agarose gel electrophoresis of inversion PCR products using DNA samples from panel A. **D,** Sanger sequencing electropherogram of the inversion breakpoint from LuCaP 105CR DNA, using the PCR product from panel C. **E,** Agarose gel electrophoresis of inversion PCR products using DNA from panel A. **F,** Sanger sequencing electropherogram of the inversion breakpoint from LuCaP 105CR DNA, using the PCR product from panel D. **G,** Agarose gel electrophoresis of inversion PCR products using from panel A. **H,** Sanger sequencing electropherogram of the inversion breakpoint from LuCaP 105CR DNA, using the PCR product from panel G. **I,** Agarose gel electrophoresis of deletion PCR products using DNA from panel A. **J,** Sanger sequencing electropherogram of the deletion breakpoint from LuCaP 105CR DNA, using the PCR product from panel I.

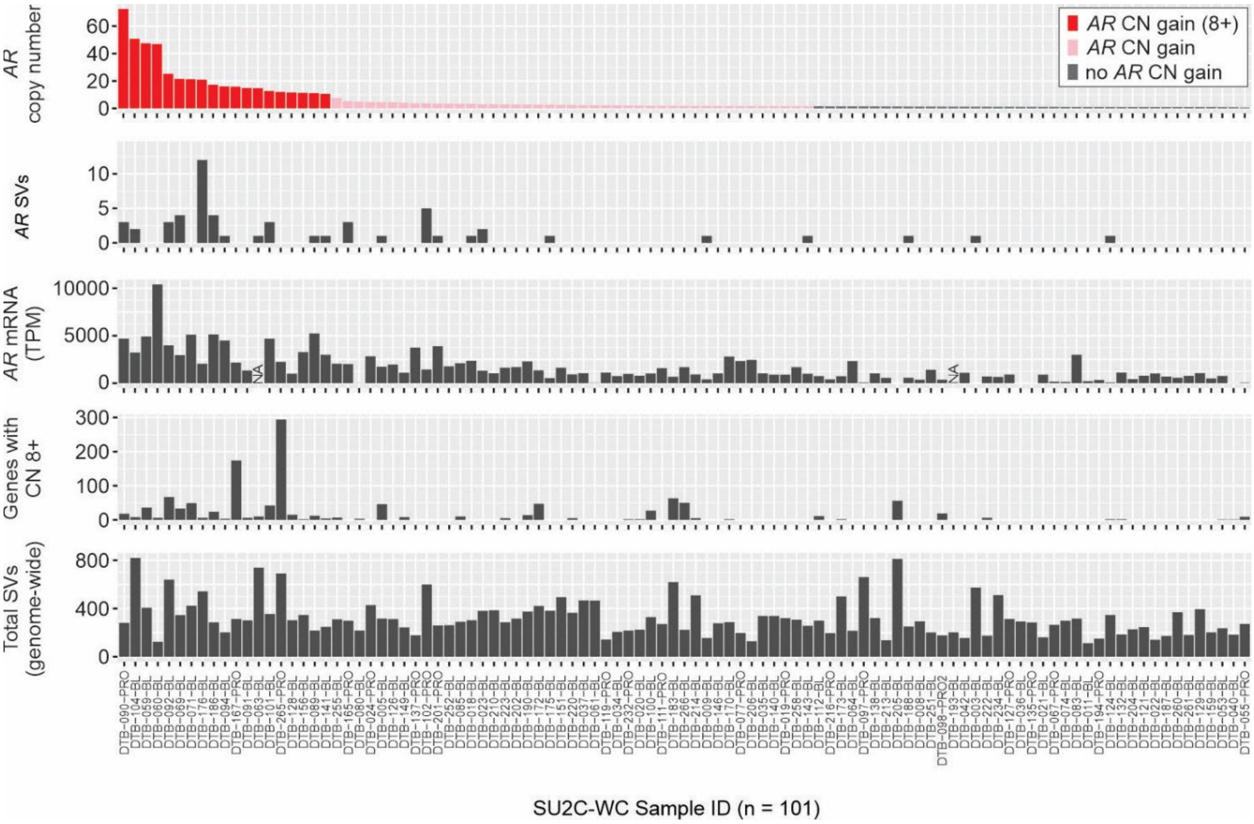

Supplementary Figure S3. Genomic context of *AR* copy number gain and *AR* gene rearrangements in clinical CRPC. Stacked plots depict *AR* copy number (CN), number of *AR* gene rearrangements (structural variants, or SVs), *AR* mRNA levels (in transcripts per million, or TPM), total number of genes with copy number (CN) of at least 8, and total number of structural variants (SVs) across 101 CRPC samples analyzed by whole-genome short-read DNA-seq by the Stand Up to Cancer West Coast (SU2C-WC) consortium. Samples are ordered left to right based on highest to lowest AR copy number.

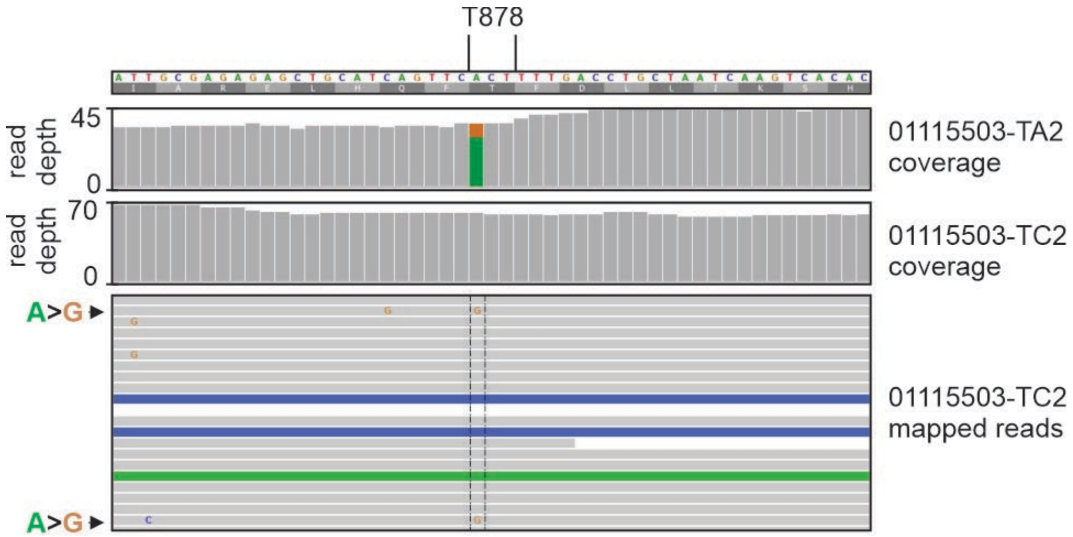

Supplementary Figure S4. DNA-seq read support of a T878A mutation in tumors TA2 and TC2 from patient 01115503. DNA-seq read coverage in tumors TA2 and TC2, with fractions of basecalls having A (green) or G (brown) alleles. Individual mapped reads are shown for sample 01115503-TC2, showing 2 reads supporting the A>G mutation.

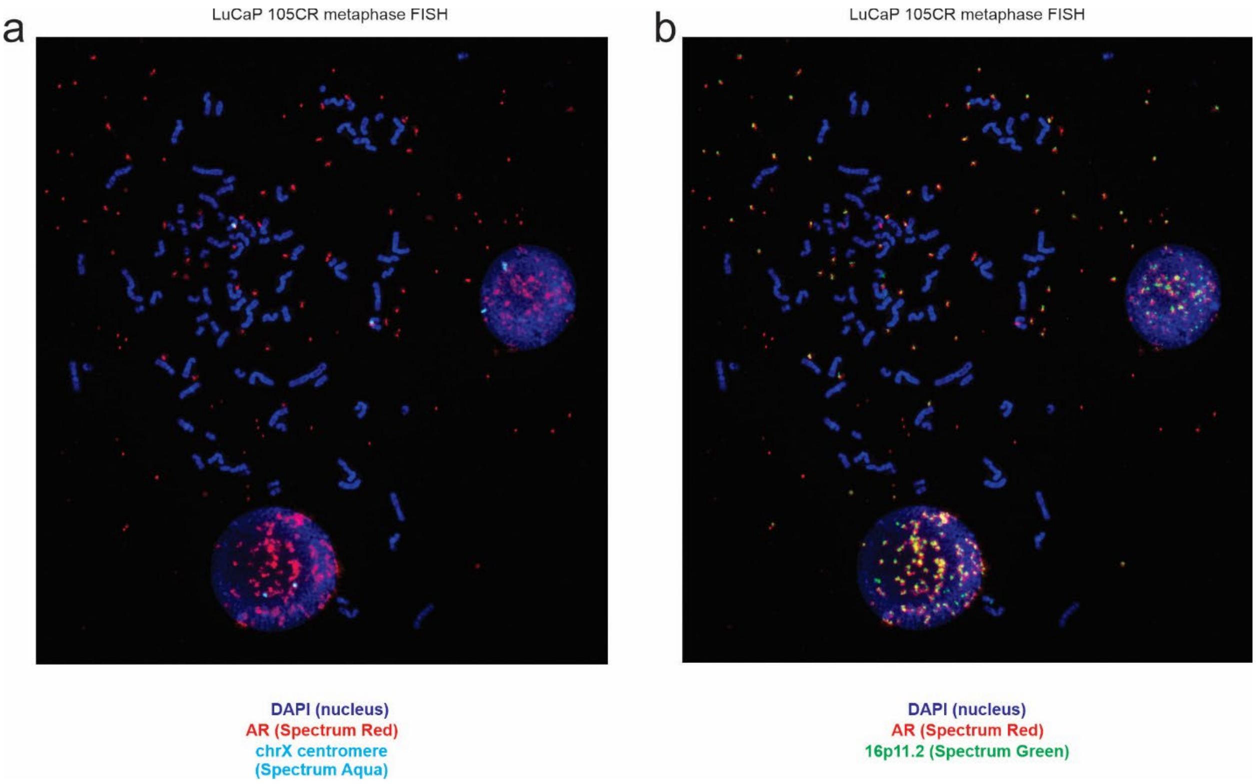

Supplementary Figure S5. AR extrachromosomal DNA (ecDNA) in LuCaP 105CR cells. Fluorescence in situ hybridization (FISH) of LuCaP 105CR in metaphase using **A,** fluorescence probes targeting AR (red) and chromosome X centromere (aqua); or **B,** fluorescence probes targeting AR (red) and 16p11.2 (green). Chromosome are stained blue with DAPI.

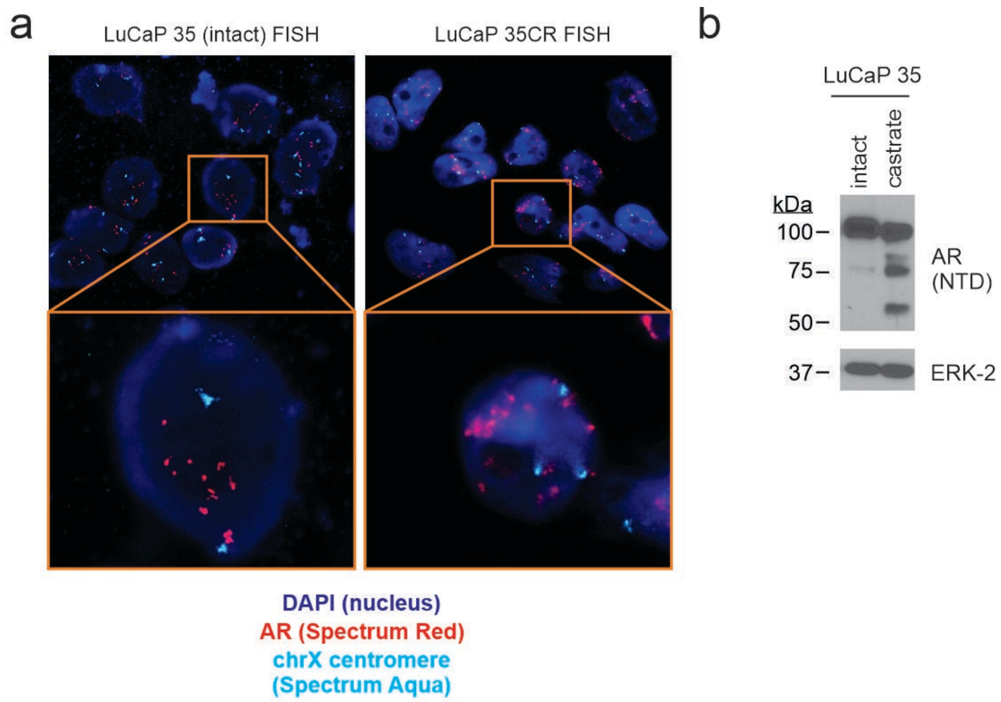

Supplementary Figure S6. ***AR*** **structural complexity evolves via eccDNA in the LuCaP 35 PDX model. A,** Fluorescence in situ hybridization (FISH) of LuCaP 35 and 35CR tumor cells using fluorescence probes targeting AR (red) and chromosome X centromere (aqua). **B,** Western blot of lysates from LuCaP 35 and LuCaP 35CR tumors with an antibody specific for the AR N-terminal domain (NTD). ERK-2 is a loading control.

Uncropped western blot films from Fig. 7b

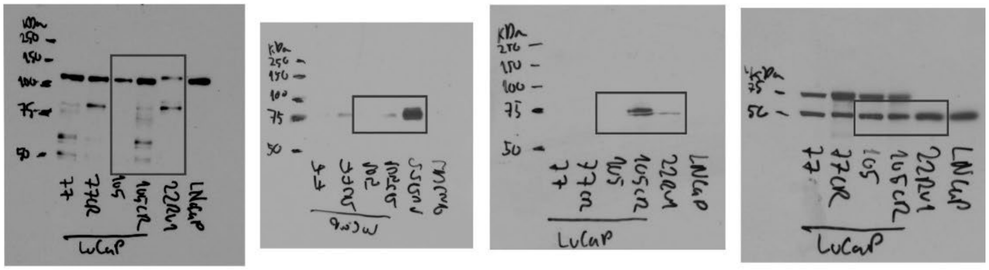

Uncropped agarose gel picture from Fig. S1b

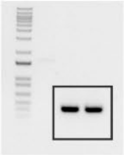

Uncropped agarose gel pictures from Fig. S2 a, c, e, g, i

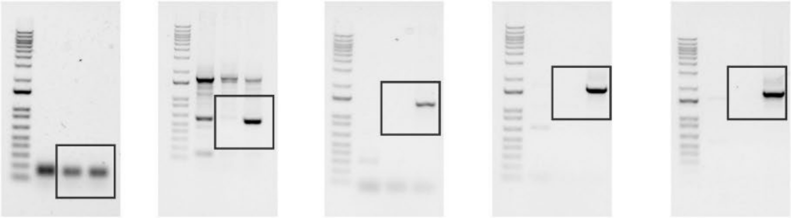

S2a S2c S2e S2g S2i

Uncropped films from Fig. S6b

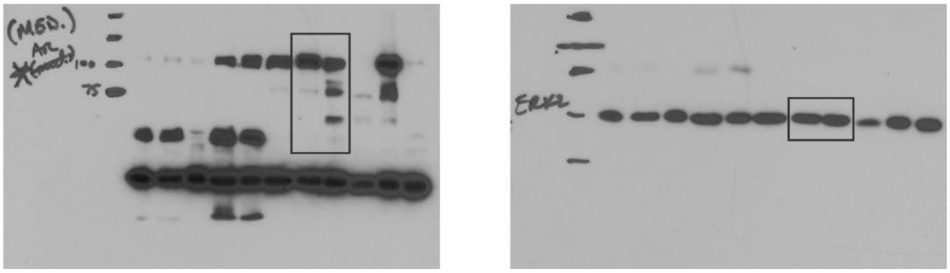

Supplementary Figure S7. Uncropped films and gels.
